# Drug repurposing of pyrazolotriazine derivatives as potential anti-SARS-CoV-2 agents: in vitro and in silico studies

**DOI:** 10.1186/s13065-024-01233-z

**Published:** 2024-07-16

**Authors:** Khulood H. Oudah, Mazin A. A. Najm, Reham F. Barghash, Omnia Kutkat, Mohamed GabAllah, Amgad Albohy, Khaled A. M. Abouzid

**Affiliations:** 1 Department of Pharmacy, Mazaya University Collage, Nasiriyah, Thi-Qar Iraq; 2 Institute of Chemical Industries Research, National Research Centre, Dokki, Giza, 12622 Egypt; 3 Faculty of Biotechnology, October University for Modern Science and Arts (MSA University), Giza, Egypt; 4 Center of Scientifc Excellence for Infuenza Viruses, National Research Centre, Dokki, Giza, 12622 Egypt; 5 Department of Pharmaceutical Chemistry, Faculty of Pharmacy, The British University in Egypt, El-Sherouk City, 11837 Cairo Egypt; 6 Pharmaceutical Chemistry Department, Faculty of Pharmacy, Ain Shams University, Abbassia, 11566 Cairo Egypt; 7 Department of microbiology, Faculty of pharmacy, Ahram Canadian University, 6 th of October, Giza, 12566 Egypt

**Keywords:** Pyrazolotriazine, COVID 19, Molecular docking, Drug repurposing, Main protease M^pro^, SARS-CoV-2

## Abstract

The search for new molecules targeting SARS-CoV-2 has been a priority since 2020. The continuous evolution of new mutants increases the need for more research in the area. One way to find new leads is to repurpose existing drugs and molecules against the required target. Here, we present the in vitro and in silico screening of ten previously synthesized and reported compounds as anti-COVID 19 agents. The compounds were screened in vitro against VERO-E6 cells to find their Cytotoxic Concentration (CC_50_) and their Inhibitory Concentration (IC_50_). Compounds **1**, **2**, and **5** revealed a promising anti-SARS-CoV-2 of (IC_50_ = 2.4, 11.2 and 2.8 µM), respectively while compounds **3** and **7** showed moderate activity of (IC_50_ = 17.8 and 26.1 µM) compared to Chloroquine which showed an IC_50_ of 24.9 µM. Among tested compounds, **1** showed the highest selectivity (CC_50_/IC_50_) of 192.8. Docking, molecular dynamics and ADME studies were done to investigate potential interactions between compounds and SARS-CoV-2 targets as well as to study the possibility of using them as lead compounds.

## Introduction

During the previous years, the world has been suffering from the newly evolving severe acute respiratory syndrome coronavirus 2 (SARS-CoV-2) [1–4]. COVID-19 first appeared in Wuhan, China, in December 2019, where the first pneumonia cases of unknown origin were recorded [5–8]. The disease has spread swiftly over the world, and on March 11th, 2020 [9], the World Health Organization (WHO) proclaimed a COVID-19 pandemic. To date, new mutants are still evolving which creates an urge for more research to be done in area of anti-SARS-CoV-2 agents.

**Fig. 1:**
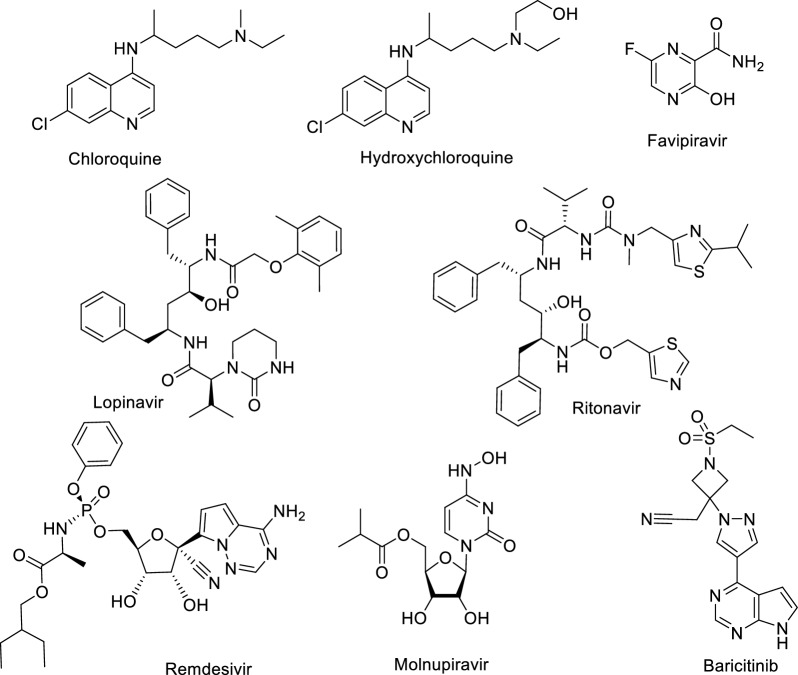
Chemical structures of some drugs repurposed against COVID-19

Until now, a number of vaccinations have been developed to stop COVID-19 pandemic from global spreading [10]. In addition, more than 9000 clinical trials related to COVID-19 are currently available on the ClinicalTrials.gov database and subjected to off-label and repurposed therapies including Chloroquine, Hydrochloroquine, Lopinavir-Ritonavir, Remdesivir, Molnupiravir, Favipiravir and Baricitinib [11–13] (Fig. 1). Additionally, significant and urgent efforts have been made to find effective treatments/drugs as potential therapies for those who had already contracted the disease.

Among drugs approved for COVID-19 treatment by the U.S. Food and Drug Administration (FDA); is a drug called Veklury (Remdesivir) [14–16], which approved in October 2020, as the first drug for the treatment of severe COVID-19 cases requiring hospitalization [16]. In November 2020 [17], Olumiant (baricitinib) was also FDA approved followed by Lagevrio (Molnupiravir) FDA approval for emergency uses authorization in December 2021 [18, 19].

Typically, it takes many years to develop or generate new drugs and evaluate them for safety and efficacy in clinical trials. The average cost of this process is close to billion dollars per drug [20]. Recent approaches towards drug discovery include using the computational structure-based drug discovery, molecular modelling and molecular simulation [13, 21–23]. Another strategy involves utilizing already approved drugs in new ways [24]. so-called drug repurposing which could be considered as one of the fastest and most affordable options to find COVID-19 treatments [25]. Compared to de novo drug development, it results in faster drug approval at a cheaper cost and shorter time [25–27]. To find new uses for already-established drugs that have successfully completed in-depth clinical trials, numerous experimental, computational drug repurposing methodologies have been developed [28–31]. Recent publications reported treating COVID-19 patients with anticancer drugs [32–35] which inspired us for this current work.

Interestingly, the pyrazolopyrimidine and pyrazolotriazine scaffolds stand out for several pharmacological activities; among which are anticancer [36–41] and antiviral [42]. In this work, we decided to follow up on ten pyrazolotriazine compounds have been previously prepared and showed a promising activity as targeted anticancer agents [41]. These compounds were tested for their cytotoxicity and SARS-CoV-2 inhibition against a strain collected and confirmed in Egypt (hCoV-19/Egypt/NRC-03/2020 (Accession Number on GSAID: EPI_ISL_430820). The potential target of these compounds was investigated using molecular modelling and dynamics studies.

## Experimental

### Chemistry

**Fig. 2:**
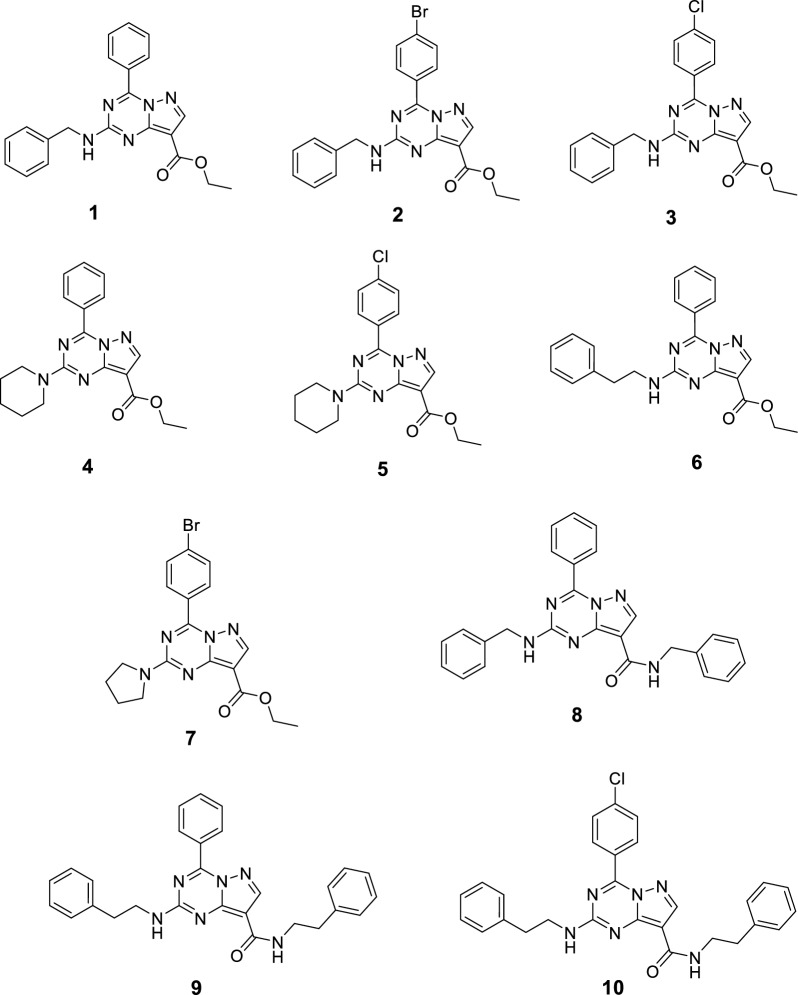
Chemical Structures of the tested compounds

The synthesis of the target substituted pyrazolotriazine derivatives (Fig. 2) was performed according to previously reported procedure [41].

### Antiviral activity

#### Compounds safety (MTT cytotoxicity assay)

The safety of the test compounds was assayed by testing their cytotoxicity using MTT method as reported earlier [43]. In brief, cytotoxicity of the test compounds was tested against VERO-E6 cells in a standard 96 well-plate. After incubation, treatment with serial concentration of the test compound, then treatment with MTT, cells were reincubated for 4 h at 37 °C. Media was collected, prepared and absorbance was measured at 540 nm using a plate reader. The absorbance obtained from treated cells was compared to that obtained from non-treated cells to calculate percent cytotoxicity. Half maximal cytotoxic concentration (CC_50_) was obtained from a plot of percent toxicity against compound concentration [44].

#### Compounds efficiency (Inhibitory concentration 50 (IC_50_) determination)

Efficiency of test compounds was tested through the determination of their IC_50_ values against VERo-E6 cells after treatment and virus absorption using SARS-CoV-2 virus obtained and confirmed in Egypt (hCoV-19/Egypt/NRC-03/2020 (Accession Number on GSAID: EPI_ISL_430820) as reported earlier [43]. Incubated cells were fixed using paraformaldehyde (4%) then stained with crystal violet. The color obtained from dissolving the dye in methanol was measured at λ max of 570 nm using Anthos Zenyth 200rt plate reader (Anthos Labtec Instruments, Heerhugowaard, Netherlands). The IC_50_ of the compound is that concentration required to reduce the virus-induced cytopathic effect (CPE) by 50%, relative to the virus control and were determined as reported [45].

### Molecular modeling study

#### Molecular docking study

Docking was done following the same protocol reported earlier [46]. In short, the proteins were downloaded from the protein data bank with their pdb codes (6LU7, 7BV2 and 6W4H) and prepared by removal of water and other small molecules. In addition, hydrogens were added and prepared proteins were saved using AutoDock tools. Ligands were constructed, converted to 3D structure and minimized using Avogadro [47]. Docking was done using Autodock Vina [48] within a cubic grid box with side of 25 Å centered on the co-crystalized ligand using exhaustiveness of 16.

#### Molecular dynamics

All atom molecular dynamics simulations were performed using GROMACS 2020.3 [49] for the selected protein–ligand complexes as reported earlier [50]. In brief, SwissParam server [51] was used for ligands parameterization while Charmm36 all-atom force field [52] was used to generate topology files for the protein. Ligand coordinates obtained from docking studies for compounds **1** and **5** were used to build complexes. Solvation was done by surrounding these compleses with dodecahedron boxes and then filling them with explicit water (TIP3P) [53]. Neutralization of the final complexes was done by adding the required number of either sodium or chloride ions. Energy minimization of the genrated solvated neutralized complexes was done using steepest descent algorithm. The complexes were then equillibrated to reach the target templerature and pressure using two successive rounds of 1 ns equillibration following NVT then NPT ensembles. One hundred nanosecond production run was performed during which the resulted trajectories were collected. Temperature was kept at 300 K during equillibration steps and production run using the V-rescale algorithm [54] while pressure was controlled using the Parrinello-Rahman barostat [55] as required. The LINear Constraint Solver (LINCS) algorithm [56] and Particle mesh Ewald (PME) method [57] were used for bond’s length constraints and long-range electrostatics calculations, respectively. All simulations were done using two femtosecond timesteps. Van der Waals distance cut-off (rvdw) was set to 1.2 nm. Trajectories collected during the production run were analyzed using the required GROMACS commands after correction of periodic boundary condition (PBC).

#### Physicochemical properties, drug likeness and ADMET prediction

In order to find compounds with the best drug-like qualities that can be turned into safe and effective medications, evaluating pharmacokinetic and physicochemical properties is a crucial stage in the drug development process. In this investigation, a number of factors including molar refractivity, partition coefficient (Log P), rotatable bonds, hydrogen bond acceptor–donor (HBA-D), and topological polar surface area (TPSA) were predicted using the SwissADME server [58]. Additionally, the ADMET properties were predicted using the pkCSM tool [59].

This tool offers comprehensive details on a number of characteristics that may affect the compound's safety and effectiveness as a prospective medication candidate. The process was carried out in accordance with accepted medicinal chemistry standards, assuring that the compounds chosen for additional research had the potential to be turned into secure and efficient medicines.

## Results and discussion

### Antiviral activity

**Fig. 3:**
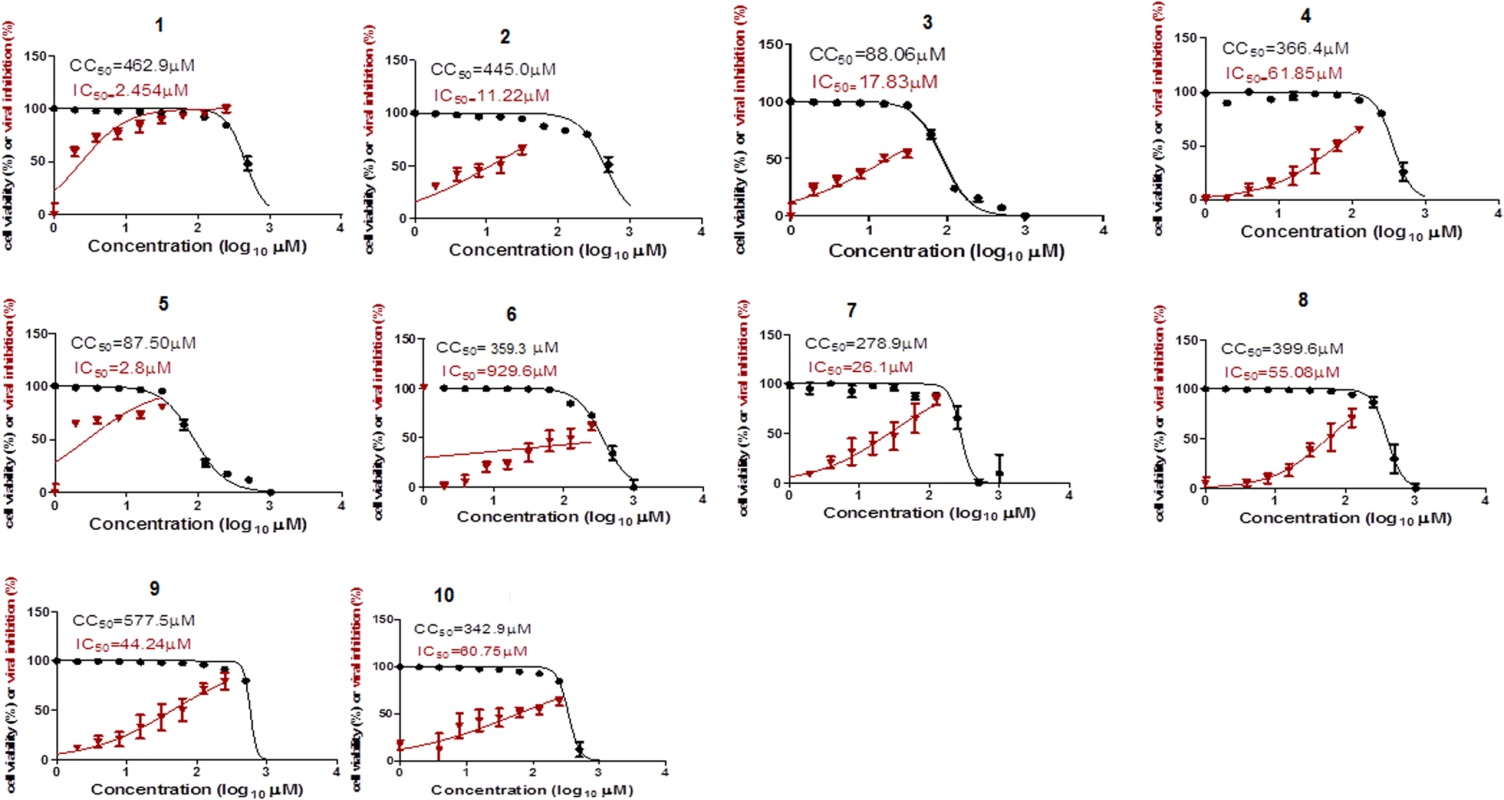
Dose–response curves for the tested compounds against SARS-CoV-2

The antiviral activity of the tested compounds were measured against SARS-CoV-2 using the standard VERO-E6 cells as it is more permissive for SARS-CoV-2 infection than airway epithelial cells, allowing virus isolation and characterization [60]. All compounds were tested on VERO-E6 without infection of the healthy cell line to detect cytotoxic concentration (CC_50_) for each compound on the cells (Fig. 3) to detect Safety Index (SI) for each one equal CC_50_/IC_50_ (Table 1), using Hydroxychloroquine and Chloroquine and Favipiravir as standard reference drug. Some of the tested compound showed good activity against SARS-CoV-2. These compounds showed promising inhibition effect against the viral propagation and infectivity of the virus compared with standard references as compounds **1**, **2**, and **5** revealed a promising anti-SARS-CoV-2 with IC_50_ = 2.4, 11.2 and 2.8 µM, respectively while compounds **3** and **7** showed moderate activities (IC_50_ = 17.8 and 26.1 µM, respectively). Repurposed drugs tested (Hydroxychloroquine, Chloroquine and Favipiravir) showed IC_50_ ranging from 24.9 to 1382 μM. The selectivity index (SI) was calculated to correlate the antiviral properties and cytotoxicity. Compound **1** showed the best SI value equal 192.8 followed by compounds **2** and **5** compared with tested standards drug as chloroquine showed best SI of 15. In general, ester derivatives at position 8 (**1–7**), showed better activity compared to amide derivatives (**8–10**). In addition, hydrophobic amino derivatives at position 2 did not affect the activity to a large extent. Furthermore, halo phenyl group at position 4 were generally more active than the unsubstituted phenyl derivatives. These results suggest that our compounds could be a promising lead as anti-COVID 19. Further investigations especially for potential SARS-CoV-2 targets are required.

**Table 1:** Cytotoxicity and virus-inhibition effect of the tested pyrazolotriazine derivatives (1–10) against SARS-CoV-2 
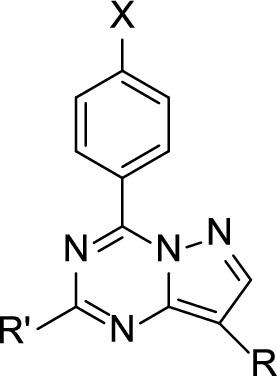

Compound	X	R	R’	IC_50_, (µM)	CC_50_, (µM)	SI^a^
1	H	COOEt		2.4	462.9	192.8
2	Br	COOEt		11.2	445.0	39.7
3	Cl	COOEt		17.8	88.0	4.9
4	H	COOEt		61.8	366.4	5.9
5	Cl	COOEt		2.8	87.5	31.2
6	H	COOEt		359.3	929.6	2.5
7	Br	COOEt		26.1	278.9	10.6
8	H	CONHCH_2_Ph		55.0	399.6	7.2
9	H	CONH(CH_2_)_2_Ph		44.2	577.5	13.0
10	Cl	CONH(CH_2_)_3_Ph		60.7	342.9	5.6
Hydroxy-chloroquine [61]			–	36.9	356.4	9.7
Chloroquine [61]			–	24.9	377.7	15.1
Favipiravir [61]			–	1382	5262	3.8
Remdesivir [62]			–	3.38	58.12	17.18

^a^ SI = CC_50_/IC_50_

### Molecular modelling studies

#### Molecular docking

**Fig. 4:**
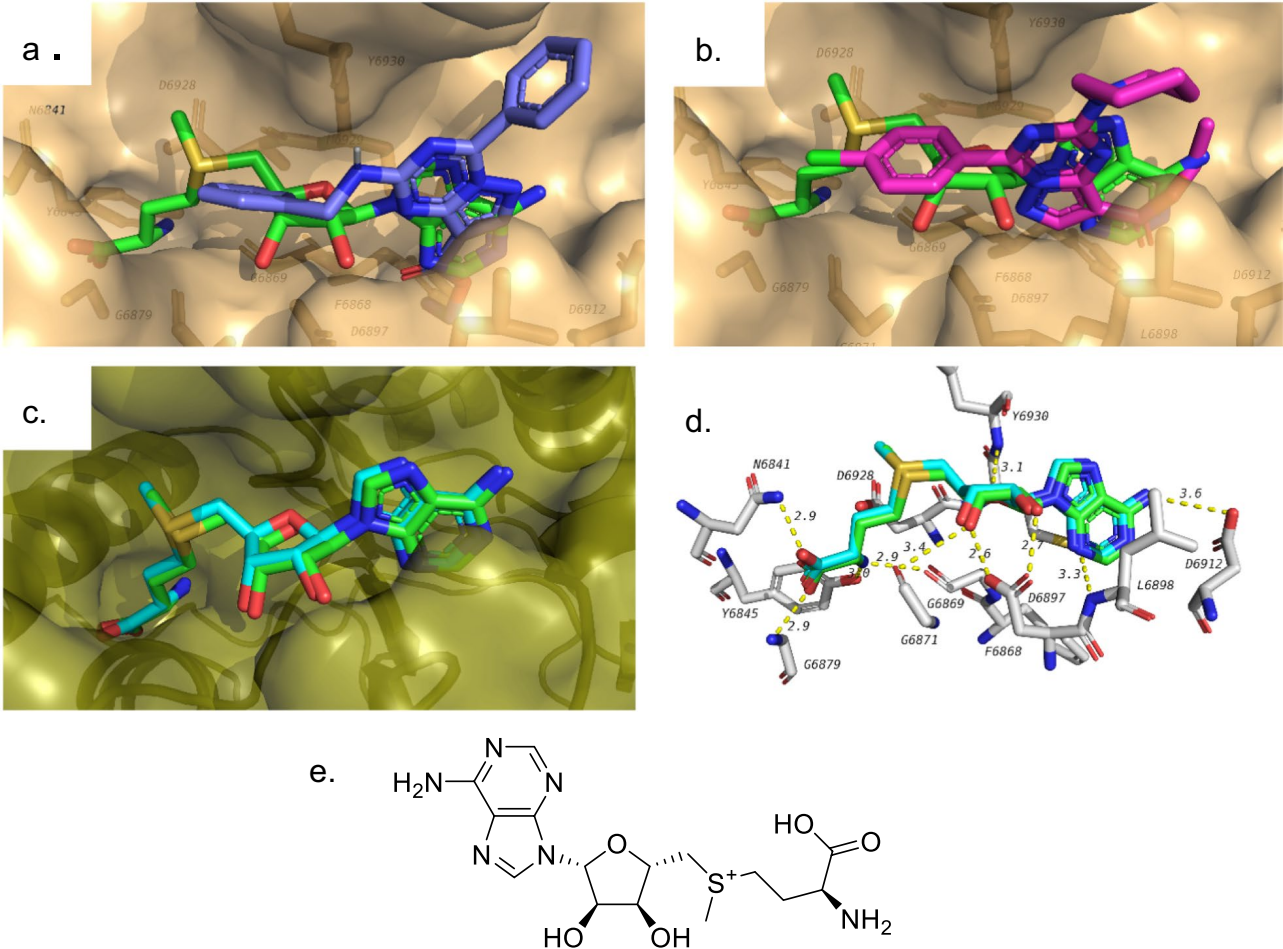
Docking results of test compounds against methyl transferase. **a** docking pose of compound 1 (dark blue) overlapped with co-crystalized ligand (green). **b** docking pose of compound 5 (pink). **c** validation of docking procedure showing docked (blue) pose overlapped with co-crystalized pose (green). **d** interactions of co-crystalized ligand in the active site methyl transferase. **e** structure of S-adenosylmethionine (SAM)

Docking was done to investigate possible SARS-CoV-2 targets. Tested compounds were docked in the active sites of three different viral proteins that include SARS-CoV-2 Main protease (M^pro^), RNA dependent RNA polymerase (RdRp) and methyl transferase. In addition to these compounds, co-crystalized ligands (N3 inhibitor, remdesivir and SAM) were docked to the active sites of their corresponding enzymes. The docking scores of each of the tested compounds in the active sites of these proteins are shown in Table 2. The most potent compounds in biological assays (**1** and **5**) showed the best docking scores with the methyltransferase with docking scores of − 8.1 and − 8.5 kcal/mol, respectively. This docking score is comparable or better than the docking score of the co-crystalized ligand in the same pdb file (6W4H). The docking poses of both compounds are shown in Fig. 4a and b, respectively. Test compounds were generally docked overlapped with the co-crystalized ligand forming similar interactions. For example, hydrogen bond with residue D6897 was maintained in both co-crystalized ligand and compound **5**. In addition, several other interactions are observed for compound 5 including hydrogen bonds with Y6930 and D6912. Also, hydrophobic interactions with F6947 and L6898 are also observed. Several of the other tested compounds also showed also docking scores better than the co-crystalized ligand as can be seen in Table 2. These results suggest that these two compounds (**1** and **5**) might have a potential inhibitory effect on this target which might require further investigations. Docking process was validated by redocking of the co-crystalized ligand (SAM, Fig. 4e) in the active site of its corresponding protein and compare its docking pose to the crystal pose. The docking procedure was accepted since the RMSD between both poses is less than 2 Å (Fig. 4c and d).

**Table 2:** Docking results of test compounds against SARS-CoV-2 targets

Compound #	6LU7	7BV2	6W4H
	M^pro^	RdRp-Mg	Methyltransferase
1	− 7.8	− 6.8	− 8.1
2	− 7.6	− 6.2	− 8.3
3	− 7.9	− 6.7	− 8.2
4	− 8.3	− 5.8	− 8.3
5	− 7.8	− 5.8	− 8.5
6	− 8	− 7.1	− 8.2
7	− 8	− 6	− 7.6
8	− 8.6	− 7.8	− 8.8
9	− 8.3	− 7.5	− 8.6
10	− 8.5	− 7.6	− 8.9
Co-crystalized Ligand	− 7.8 (N3 inhibitor)	− 6.9 (Remdesivir)	− 8.1 (SAM)

### Molecular dynamics

**Fig. 5:**
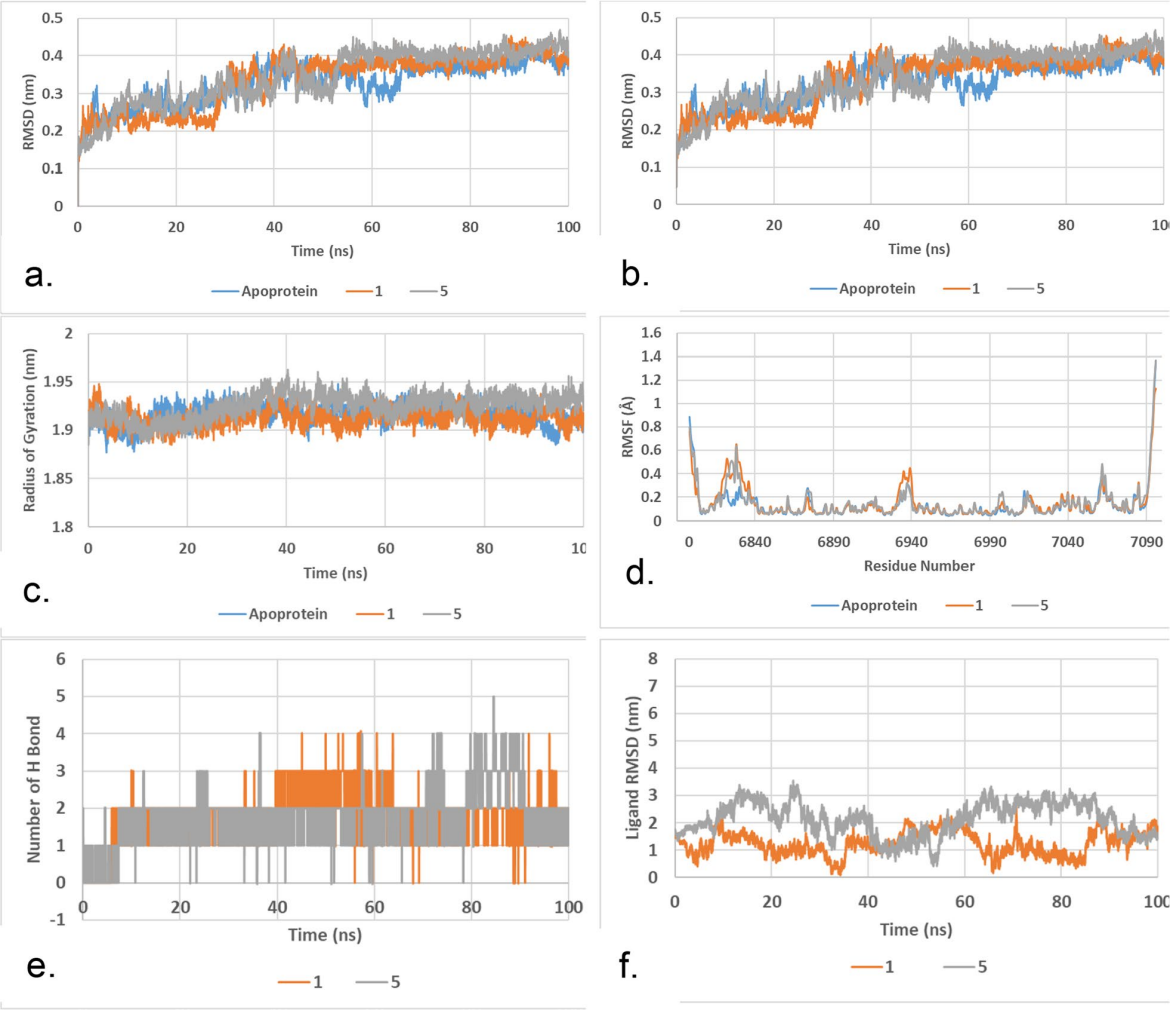
Molecular Dynamics results for compounds 1 and 5 in the active site of SARS-CoV-2 methyl transferase. **a** RMSD of protein chain relative to first frame of production run. **b** RMSD of protein chain relative to starting crystal structure. **c** Radius of gyration of protein. **d** average RMSF of protein amino acids during the production run. **e** number of hydrogen bonds between ligands and protein. **f** RMSD of ligand heavy atoms during production run

Molecular dynamic study was performed to follow up on the docking results and to investigate the stability of **1** and **5** in the methyl transferase active site of SARS-CoV-2 (6W4H). Each complex along with the apoprotein were enclosed in a dodecahedron boxe which were filled with water and ions to simulate experimental condition. After minimization, the systems were equilibrated, and temperature was adjusted to 300 ˚K and pressure was adjusted for 1 atm. Then complexes were subjected to 100 ns of production run at the same temperature and pressure. Collected trajectories were analyzed to extract information about these complexes compared to the apoprotein. The results are shown in Fig. 5 and Table 3.

**Table 3:** Results of molecular dynamics study

	Apoprotein	1	5
RMSD (nm)	0.327 ± 0.056	0.334 ± 0.073	0.344 ± 0.073
RMSD_Crystal (nm)	0.327 ± 0.056	0.334 ± 0.072	0.343 ± 0.072
Radius of gyration (nm)	1.916 ± 0.010	1.913 ± 0.010	1.925 ± 0.012
Number of H bonds	–	1.97 ± 0.71	1.89 ± 0.79
RMSD of ligand (nm)	–	1.254 ± 0.436	2.133 ± 0.610

Root mean square deviation (RMSD) of protein relative to first frame and to crystal structure (Fig. 5a and b) shows very similar results for the apoprotein as well as the two studies complexes. The fluctuation of RMSD value (Table 3) is less than 1 Å which indicates the stability of the protein during the production run. This also shows that the insertion of these two inhibitors does not affect the protein structure. Furthermore, radius of gyration (Fig. 5c), which is a measurement of protein compactness, was very stable during the production run which also support the above finding. Figure 5d shows a plot of the root mean square fluctuation (RMSF) of protein individual residues during the production run. The high similarity between all the complexes as well as the small values (mostly less than 1 Ǻ except for terminal residues) supports the stability of protein and the protein–ligand complexes. The last 2 plots (Fig. 5e and f) compare the interactions of ligands 1 and 5 with target protein. as seen in the figures as well as Table 3, compound 1 is predicted to have better interaction with the target protein as it shows higher average of hydrogen bonds formed. In addition, compound 1 showed less RMSD value (1.25 Å versus 2.13 Å for compound 5) as well as less fluctuation compared to compound 5 (0.44 Å for compound 1 versus 0.61 Å for compound 5). These results indicate the higher stability and tighter binding of compound 1 compared to compound 5 in the active site of target protein. In addition, energy calculation for the interactions of compounds 1 and 5 with the target protein shows that both compounds showed similar short range coulomb interaction with the target protein (− 216.111 ± 16 kJ/mol for compound 1 versus − 214.82 ± 24 kJ/mol). On the other hand, compound 1 showed significantly better short range Lennard–Jones (L-J) interaction with target (− 27.1232 ± 5 kJ/mol for compound 1 versus -15.3226 ± 4 kJ/mol for compound 5). These findings suggest the importance of the side chain of compound 1 (phenyl methyl amine) in the formation of hydrophobic interactions with the target protein which reflects the improved L-J interaction energies without affecting coulombic interaction. These results opens the door to further follow up studies to confirm these interactions and use them to improve inhibition of this important target against SARS-CoV-2 virus.

#### Physicochemical, drug-likeness properties:

We also were interested in examining the drug-likeness of compounds by the SwissADME server [58]. When determining whether a compound has a chance to develop into a drug, a set of criteria referred to as drug-likeness properties are used. Molar refractivity, molecular weight, lipophilicity (log P), HBA, HBD, the number of rotatable bonds, and TPSA are some of the descriptors used to evaluate these qualities. One of these is Lipinski's rule [63] of five, which emphasizes that a compound is more likely to be orally bioavailable if it meets certain criteria, such as having a molecular weight of less than 500, a log P of less than 5, no more than five H-bond donors and no more than ten H-bond acceptors.

The physicochemical parameters acquired from the SwissADME server (Table 4) are within the acceptable ranges, indicating that the tested compounds **1–10** fully complied with Lipinski's rules [63]. This indicates that they possess advantageous characteristics for example optimal size, flexibility, as well as polarity that are associated with bioavailability. The ADME prediction (Table 5) reveals that all of the compounds respond to other essential drug-likeness rules, including those of *Ghose *et al. and *Muegge *et al. [64–67], with the exception of compounds **9** and **10**, which have only one variable for these two rules. In addition, the analyzed substances exhibited excellent bioavailability scores of 0.55. These results suggests that these previously synthesized compounds have significant potential as drug candidates and could be investigated further for their therapeutic properties, according to the findings.

**Table 4:** Predicted physicochemical properties of compounds 1–10 using the SwissADME server

Compound	Molecular weight (< 500)	TPSA^a^ (< 140A˚^2^)	H-Bond Acceptor (< 10)	H-Bond Donnor (< 5)	LogP (< 5)	Lipinski’sViolation
1	373.41	81.41	5	1	3.32	0
2	452.3	81.41	5	1	3.93	0
3	407.85	81.41	5	1	3.85	0
4	351.4	72.62	5	0	2.93	0
5	385.85	72.62	5	0	3.39	0
6	387.43	81.41	5	1	3.58	0
7	416.27	72.62	5	0	3.28	0
8	434.49	84.21	4	2	3.88	0
9	462.55	84.21	4	2	4.34	0
10	496.99	84.21	4	2	4.88	1

^a^Topological polar surface area

**Table 5:** Drug-likeness properties of compounds 1–10 by the SwissADME server

Compound	Lipinski	Ghose	Veber	Egan	Muegge	Bioavailability score
1	Yes	Yes	Yes	Yes	Yes	0.55
2	Yes	Yes	Yes	Yes	Yes	0.55
3	Yes	Yes	Yes	Yes	Yes	0.55
4	Yes	Yes	Yes	Yes	Yes	0.55
5	Yes	Yes	Yes	Yes	Yes	0.55
6	Yes	Yes	Yes	Yes	Yes	0.55
7	Yes	Yes	Yes	Yes	Yes	0.55
8	Yes	Yes	Yes	Yes	Yes	0.55
9	Yes	No	Yes	Yes	No	0.55
10	Yes	No	Yes	Yes	No	0.55

**Fig. 6:**
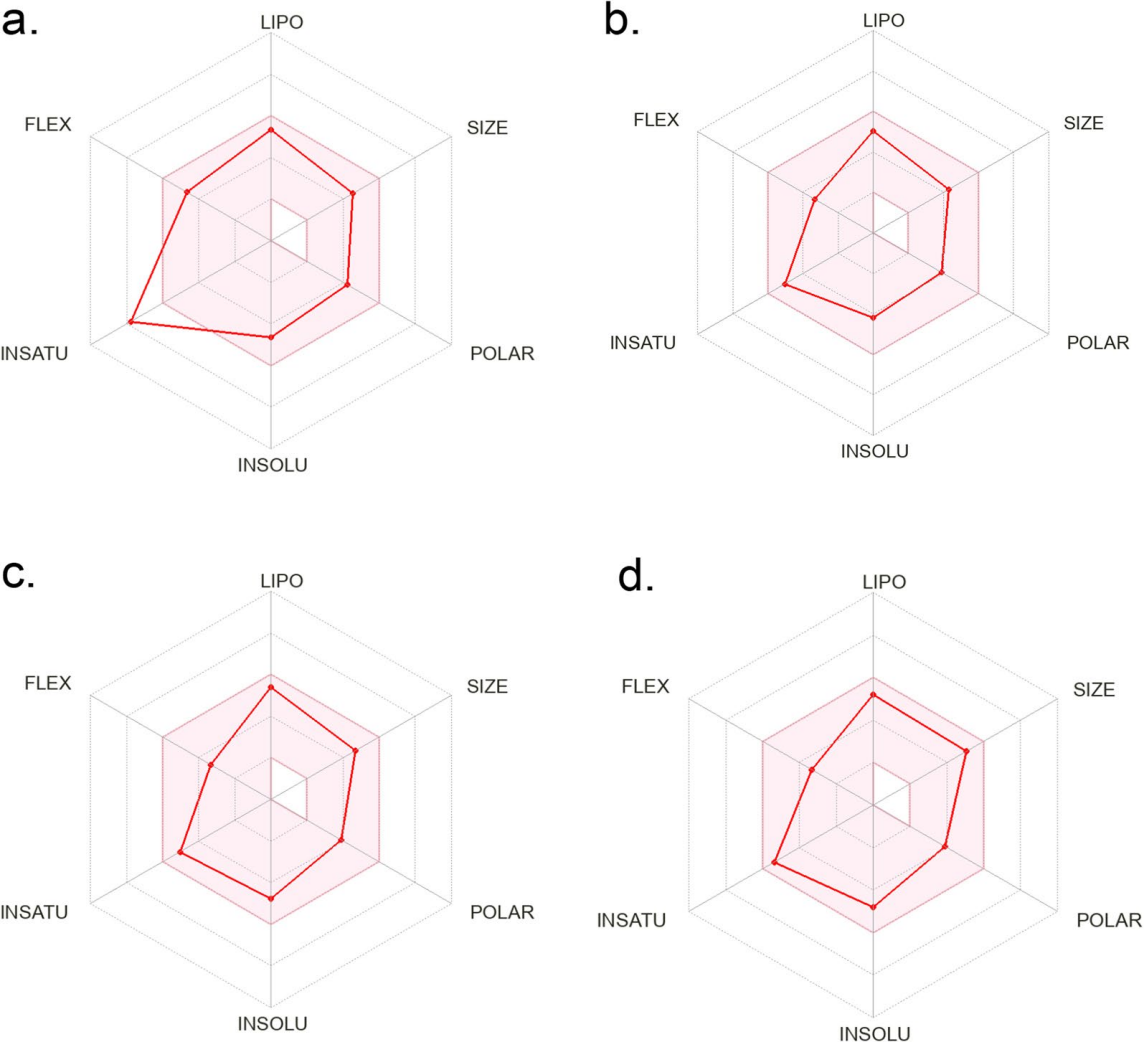
Bioavailability radar for compounds 1 (**a**), 4 (**b**), 5 (**c**) and 7 (**d**)

In addition, the SwissADME server was used for plotting the bioavailability radar of compounds (Fig. 6) which is also another way to represent parameters affecting bioavailbility. Figure 6 shows representative examples for the bioavailability radar for 4 compounds including **1**, **4**, **5** and **7**. All parameters for all compounds are within the acceptable ranges except insaturation of compound **1**, which was slightly higher.

#### ADMET properties of the investigated compounds

ADMET (Absorption, distribution, metabolism, elimination and toxicity) properties are necessary to assess a prospective drug candidate's compatibility for clinical application. Along with being effective at low concentrations and having a low degree of toxicity, a drug candidate must have sufficient pharmacokinetic properties which maintain the drug molecules available in their active state for the duration of action desired. The computational assessment of ADMET properties can substantially assist the prediction of the effects of a drug candidate in the human system, thereby accelerating and streamlining the process of developing drugs and reducing the risk of collapse during clinical trials. Therefore, a comprehensive evaluation of the characteristics of ADMET is essential for the successful development and commercialization of new pharmaceuticals.[59].

According to data in Table 6, tested compounds showed medium solubility in aqueous media as well as significant oral absorption (92.206%–99.129%), ensuring exceptional absorption which is an essential requirement for oral drugs. In addition, this finding is supported by the predicted Caco2 permeability values indicating the suitability of the oral route of administration for all tested compounds with the exception of compounds **8, 9**, and **10**. Furthermore, these compounds also showed superior skin permeability. Moreover, tested compounds are inhibitors of P-glycoproteins which could help with efflux problem seen with some inhibitors leading to the decrease in their bioavailability [68, 69]. In terms of distribution, the tested compounds have a low volume of distribution at steady state (VDss) and most of drugs were predicted to be in the bound state which means that these molecules have limited distribution which indicates the need for lower loading doses. In addition, their Blood–Brain Barrier (BBB) and the central nervous system (CNS) permeability assessments are moderate to poor, indicating the lower potential to cause CNS adverse effects [70].

**Table 6:** In silico ADMET predictions of the compounds 1–10

Property and Model Name	1	2	3	4	5	6	7	8	9	10
Absorption										
Water solubility (log mol/L)	− 4	− 4.271	− 4.229	− 3.124	− 3.578	− 4.307	− 3.453	− 4.232	− 4.44	− 4.525
Caco2 permeability (log Papp in 10-6 cm/s)	1.116	1.125	1.129	1.408	1.42	1.172	1.408	0.613	0.64	0.661
Intestinal absorption in humans (% Ab)	95.659	94.098	94.165	99.129	97.635	95.631	97.957	93.208	93.7	92.206
Skin permeability (log Kp)	− 2.754	− 2.756	− 2.756	− 2.735	− 2.765	− 2.757	− 2.761	− 2.735	− 2.735	− 2.735
P-glycoprotein substrate (Yes/No)	No	No	No	No	No	No	No	Yes	Yes	Yes
P-glycoprotein I inhibitor (Yes/No)	Yes	Yes	Yes	Yes	Yes	Yes	Yes	Yes	Yes	Yes
P-glycoprotein II inhibitor (Yes/No)	Yes	Yes	Yes	No	Yes	Yes	Yes	Yes	Yes	Yes
Distribution										
VDss in humans (log L/kg)	0.021	0.091	0.073	− 0.128	− 0.107	0.062	− 0.146	0.09	0.107	0.158
Fraction unbound in humans (Fu)	0.042	0.045	0.047	0.123	0.13	0.003	0.139	0.096	0.067	0.061
BBB permeability (log BB)	− 0.465	− 0.646	− 0.638	− 0.872	− 1.061	− 0.42	− 1.074	0.023	0.113	− 0.059
CNS permeability (log PS)	− 2.477	− 2.335	− 2.358	− 2.539	− 2.975	− 2.49	− 2.992	− 2.083	− 2.208	− 2.097
Metabolism										
CYP2D6 substrate (Yes/No)	No	No	No	No	No	No	No	No	No	No
CYP3A4 substrate (Yes/No)	Yes	Yes	Yes	Yes	Yes	Yes	Yes	Yes	Yes	Yes
CYP1A2 inhibitor (Yes/No)	Yes	Yes	Yes	Yes	Yes	Yes	Yes	Yes	Yes	Yes
CYP2C19 inhibitor (Yes/No)	No	No	No	No	No	Yes	No	Yes	Yes	Yes
CYP2C9 inhibitor (Yes/No)	No	No	No	No	Yes	Yes	Yes	Yes	Yes	Yes
CYP2D6 inhibitor (Yes/No)	No	No	No	No	No	No	No	No	No	No
CYP3A4 inhibitor (Yes/No)	Yes	Yes	Yes	No	No	Yes	No	Yes	Yes	Yes
Excretion										
Total Clearance (log ml/min/kg)	0.358	0.185	0.092	0.387	0.101	0.388	0.242	0.128	0.191	− 0.078
Renal OCT2 Substrate (Yes/No)	No	No	No	No	No	No	No	No	No	No
Toxicity										
AMES toxicity (Yes/No)	No	No	No	Yes	No	No	No	Yes	Yes	No
Max. tolerated dose in humans (log mg/kg/day)	0.35	0.272	0.271	− 0.044	− 0.047	0.324	− 0.031	0.697	0.628	0.622
hERG I inhibitor (Yes/No)	No	No	No	No	No	No	No	No	No	No
hERG II inhibitor (Yes/No)	Yes	Yes	Yes	Yes	Yes	Yes	Yes	Yes	Yes	Yes
Oral Rat Acute Toxicity (LD50) (mol/kg)	3.318	3.268	3.264	2.542	2.534	3.189	2.5	3.098	2.917	2.894
Oral Rat Chronic Toxicity (LOAEL) (log mg/ky_bw/day)	1.207	1.021	1.031	0.89	1.041	1.259	1.014	1.271	1.354	1.291
Hepatotoxicity (Yes/No)	Yes	Yes	Yes	Yes	Yes	Yes	Yes	Yes	Yes	Yes
Skin sensitization (Yes/No)	No	No	No	No	No	No	No	No	No	No
*T. Pyriformis* toxicity (log ug/L)	0.295	0.295	0.295	0.311	0.31	0.301	0.311	0.285	0.285	0.285
Minnow toxicity (log mM)	− 2.137	− 2.544	− 2.398	0.097	− 0.443	− 3.214	− 0.472	0.082	− 0.953	− 1.2

The tested compounds were predicted to interact with CYP3A4 which is one of the liver microsomal enzymes involved in the metabolism of various drugs. All compounds were predicted to be both substrates and inhibitors except compounds **4, 5**, and **7** which are substrates but not inhibitors. These compounds, however, are not substrates for another variant which is CYP2D6. This might be an advantage, as this enzyme is known to be involved in the metabolism of several known drugs. In terms of elimination, tested substances showed a medium clearance and are not predicted to be renal OCT2 substrates [71].

Furthermore, the compounds that were examined showed signs of hepatotoxicity but did not cause skin sensitization. Moreover, the AMES toxicity test was negative for the majority of the compounds, which suggests that the examined compounds are not mutagenic, with the exception of compounds **4, 8,** and **9**. These three compounds showed positive results. Tested compounds do not inhibit hERG I, but they exhibit a certain inhibitory effect on hERG II, known to be involved in some cardiac arrhythmias [72]. This is in contrast to hERG I, which they were not predicted to inhibit. The elevated human tolerated dose (0.271–0.697 log mg/kg/day) also serves as a good indication for the reasonably safe profile of these compounds, with the exception of compounds **4, 5**, and **7**.

In general, these ADMET predictions are able to provide information that can be helpful when further assessing the potential of the compounds that have been investigated as therapeutic candidates. In addition to the predictions made using computer models, the real-life pharmacokinetic characteristics of the drug candidate need to be tested in vitro as well as in vivo in order to validate the efficacy and safety of the pharmaceutical approach [72, 73].

## Conclusion

In this work we report the screening of ten previously reported compounds to repurpose them against SARS-CoV-2. Among tested compounds 3 compounds showed promising results as viral inhibitors with IC_50_ values 2.4–11.2 μM. Compound **1** showed best inhibition and has the best selective index with CC_50_ 190-fold larger than IC_50_. It worth to mention that all top compounds have the smaller ethyl carboxylate substitution at position 8 on the pyrazolotriazine ring. In addition, two of the tested compounds had 4-halophenyl substitution which suggest the priority of these function group. Docking studies suggests that these compounds have a potential effect on methyl transferase. Follow up molecular dynamics study was also performed to investigate the stability of compounds **1** and **5** in the active site of SRS-CoV-2 methyl transferase and suggested that compound **1** has the highest potential against this important target. The results of this study suggest that these compounds should be further investigated as leads against SARS-CoV-2 targets especially methyl transferase.

## Data Availability

The datasets used and/or analysed during the current study are available from the corresponding author on reasonable request.
